# Re-usable self-poled piezoelectric/piezocatalytic films with exceptional energy harvesting and water remediation capability

**DOI:** 10.1016/j.nanoen.2020.105339

**Published:** 2020-12

**Authors:** Biswajoy Bagchi, Nur Amin Hoque, Norbert Janowicz, Sukhen Das, Manish K. Tiwari

**Affiliations:** a Wellcome/EPSRC Centre for Interventional and Surgical Sciences (WEISS), University College London, London, W1W 7TS, UK; b Nanoengineered Systems Laboratory, UCL Mechanical Engineering, University College London, London, WC1E 7JE, UK; c Jadavpur University, Department of Physics, Kolkata, 700032, India

**Keywords:** MoS_2_ nanoflower, PVDF, Self-poling, Nanogenerator, Piezocatalysis, Water remediation

## Abstract

The need for sustainable technologies to address environmental pollution and energy crisis is paramount. Here we present a novel multifunctional nanocomposite, free standing film by combining piezoelectric molybdenum sulphide (MoS_2_) nanoflower with poly vinylidene fluoride (PVDF) polymer, which can harness otherwise wasted mechanical energy for useful energy generation and/or water purification. The unique MoS_2_ nanoflower morphology is exploited to render the whole nanocomposite piezo active. A number of features are demonstrated to establish potential practical usage. Firstly, the nanocomposite is piezoelectric and piezocatalytic simultaneously without requiring any poling step (i.e. self-poled). Secondly, the self-poled piezoelectricity is exploited to make a nanogenerator. The nanogenerator produced >80 V under human finger tapping with a remarkable power density, reaching 47.14 mW cm^−3^. The nanocomposite film is made by simple solution casting, and the corresponding nanogenerator powers up 25 commercial LEDs by finger tapping. Last but not the least, the developed films show efficient, fast and stable piezocatalytic dye degradation efficiency (>90% within 20 min) against four different toxic and carcinogenic dyes under dark condition. Reusability of at least 10 times is also demonstrated without any loss of catalytic activity. Overall, our nanocomposite has clear potential for use as self-powered sensor and energy harvester, and in water remediation systems. It should potentially also be deployable as a surface mounted film/coating in process engineering, industrial effluent management and healthcare devices systems.

## Highlights

• MoS_2_ nanoflower embedded self-poled PVDF nanocomposite film synthesized by simple solution casting.
• The nanocomposite film exhibited both piezoelectric (nanogenerator) and piezocatalytic activity.
• Nanogenerator feature a power density of 47.14 mW/cm^−3^ under human finger tapping.
• The films degrade harmful dyes in dark due to piezocatalytic activity.

## Graphical abstract

**Figure:**
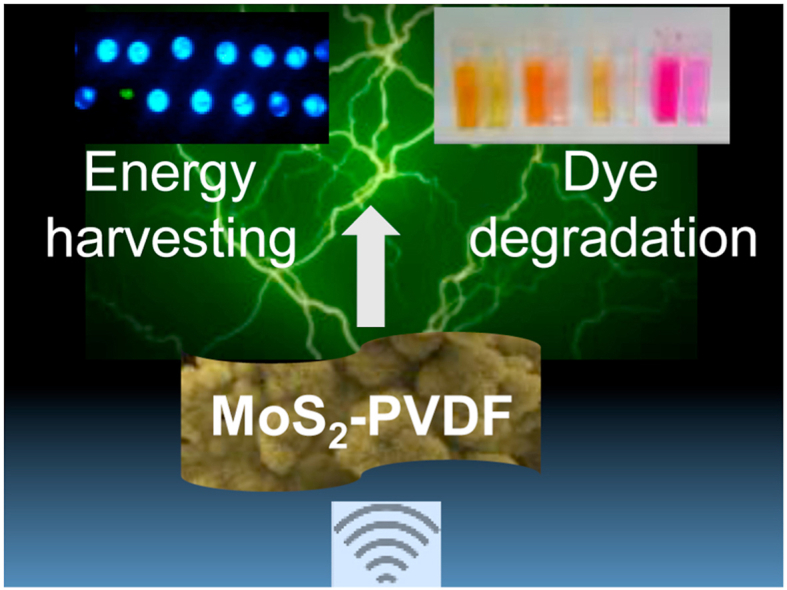

## 1 Introduction

Bioaccumulation of emerging inorganic and organic contaminants such as toxic dyes, pharmaceuticals, and drugs in wastewater effluents, natural environment and drinking waters has grown at an alarming rate in recent times and presents a huge challenge for water reuse industry [1,2]. Water pollutants such as common pesticides, herbicides, organic dyes and biological contaminants such as DNA, proteins, lipids, bacteria, virus, spores etc., present a serious and persistent threat to human health. This has prompted the development of some advanced disinfection and water remediation techniques which involves reactive oxygen species (ROS). ROS consisting of a medley of strong oxidizing agents like OH•, H•, O•, O_3_, H_2_O_2_ etc. That can safely and effectively destroy a large number non-living and living organic contaminants in water. Among the contaminants, organic dyes are particularly challenging to decontaminate since most dyes used in textile, pharmaceutical, food industries and agriculture are not only toxic to the environment but also tend to be potentially carcinogenic. Thus, complete breakdown and/or removal of these contaminants in industry effluents as well as drinking water is absolutely essential for long term benefit to human health and environment [[2], [3], [4], [5]]; and doing so in a safe and energy-efficient manner is a major scientific challenge. Current ROS based water treatment technologies such as UV photolysis, radiolysis, ozonation, sonochemistry, electrical discharge technology, electron-beam irradiation and supercritical water oxidation require expensive set up and continuous energy source in the form of electricity or ultraviolet light source [4,6,7]. Although, photocatalysis has shown promise but is limited by agglomeration tendency, post-separation difficulty and low efficiency under solar irradiation [8]. Here we present a *passive* (i.e. without requiring any additional energy input), effective, cheap and eco-friendly processes for ROS mediated disinfection and degradation of dyes using a novel piezocatalytic nanocomposite. As an additional benefit, we also demonstrate the ability of our nanocomposite to serve as a nanogenerator for energy-harvesting applications, thereby offering a promise for self-powered sensing and optoelectronic devices.

Piezoelectric materials can produce electric charge when subjected to mechanical stress and vice-versa [8,9]. Mechanically straining a piezoelectric material induces an electric field throughout the material which generates free charges at its interface with the ambient (e.g. air or water). Piezocatalytic materials are a subclass of piezoelectric materials, where the material composition is engineered such that the free charges in turn generate several reactive oxygen species (ROS) including ^•^OH, ^•^O^−^, ^•^HO_2_ and H_2_O_2_ by local micro-electrolysis of water. The ROS generated by mechanical actuation of piezoelectric materials thus can be efficiently used to piezocatalytically oxidize and degrade the toxic and/or carcinogenic dyes and microbes in water from textile, chemical, pharmaceutical and food industries [[10], [11], [12], [13], [14]].

These piezoelectric materials can also be activated when subject to ambient vibrations which are abundant in daily life. For example, piezoelectric ceramics in the form of ZnO and Cu/ZnO nanowires, Pb(Zr_0.52_Ti_0.43_)O_3_ fibers, ZnSnO_3_ nanowires, single/few-layers MoS_2_ nanoflowers and nano/micrometer sized BaTiO_3_, etc. have all been reported to piezocatalytically degrade potentially harmful dyes e.g. Acid Orange 7, Rhodamine B, Methyl Orange, 4-chlorophenolunder ultrasound vibrations with high efficiency [11,[15], [16], [17], [18], [19], [20], [21]]. Thus, harnessing the abundant vibrational and otherwise wasted mechanical energy to piezocatalytically treat waste-water can truly boost water purification technology in an energy-efficient manner. However, using free piezoelectric nanoparticles directly in water treatment presents several problems in the form of stability of the nanoparticles, efficacy, accumulation, toxicity, separation and recyclability. Here we propose a flexible, polymer nanocomposite film as an alternative solution to overcome these limitations. Our hybrid piezoelectric films are based on PVDF and MoS_2_ nanoflowers, which can be processed simply through solution processing and demonstrate excellent piezoelectric properties without requiring energy intensive poling – our nanocomposite are self-poled. The mechanism of self-poling is discussed in detail and the functionality is established by demonstrating that our films can also be used in energy-harvesting applications, such as the so called nanogenerators for self-powered sensing systems [22]. To demonstrate the dual-use feature, we exploit vibration induced electromechanical coupling to promote remarkable energy harvesting capability (demonstrated by fabricating nanogenerator device which is able to power up LEDs by human finger tapping) as well as catalytic degradation of potentially toxic and carcinogenic dyes like, Acridine Orange [23], Eosin Y [24], Ethidium Bromide [25] and Rhodamine B [26].

The piezocatalytic activity in MoS_2_ nanoflowers was first reported by Wu et al. [11]*,* who showed ultrafast degradation of Rhodamine B in water under dark condition. However, for practical application, the process runs the risk of contaminating water with MoS_2_ nanoparticles itself. Further, it is not suitable for treating flowing water due to loss of nanoparticles over time. Thus, a self-sustained system whereby the MoS_2_ nanoflowers are held stationary is required for long term application. PVDF polymer has been well known for its robustness, chemical inertness and piezoelectricity, which therefore would be an ideal candidate to act as a substrate for arresting MoS_2_ nanoflowers. The MoS_2_-PVDF based nanocomposites reported so far mainly focussed on energy harvesting applications rather than dye degradation, for example, Cai et al. [27], studied mainly MoS_2_ nanosheet induced β phase transformation and mechanical aspects of PVDF films. Maity et al. [28], showed nanogenerator and sensing performance with salt exfoliated bulk MoS_2_ and electrospun PVDF composite. Similarly, Sahatiya et al. [29], used electrospun PVDF and MoS_2_-cellullose for hybrid piezo-triboelectric nanogenerator. However, in both the applications, electrically poled PVDF nanofibers were used and no piezocatalytic properties were reported.

In the current work we are introducing a number of new features. For the first time, we are introducing a self-poled, deployable MoS_2_ nanoflower doped PVDF film, which demonstrates excellent piezoelectric (through an energy harvesting nanogenerator) and piezocatalytic functions. The unique strategy of combining MoS_2_ nanoflowers in PVDF matrix results in a multifunctional, robust, nontoxic and flexible film where the enhanced piezoelectricity is achieved by synergistic interaction between MoS_2_ and PVDF without requiring any high voltage poling process [28,30] while the piezocatalytic effect is observed due to vibration induced ROS generation at the water/film interface. The nanogenerator developed using our self-poled MoS_2_-PVDF film exhibited much superior performance compared to similar nanocomposites and repeatable multi-dye degradation capability. The simple solution casting approach to nanocomposite film synthesis is scalable and economical, and the embedded MoS_2_ nanoparticles offer the potential for long term activity, recyclability and the ability to treat large volumes of water without any risk of nanoparticle spillage. Additionally, use of a nanocomposite film instead of simply using the MoS_2_ nanoflower for piezocatalytic water remediation means that there will be no need to remove the nanoparticles. Therefore, PVDF and MoS_2_ nanoflower have a complementary role in the reported nanocomposite functionality. Notably, our approach is different from recent work by Lin et al. [31] who blended nanoflower MoS_2_ with poly(dimethyl siloxane) (PDMS) to obtain piezocatalytic behaviour and also used the nanocomposite to develop a *triboelectric* nanogenerator; our focus is on the self-poling, piezoelectric feature imparted to PVDF by MoS_2_ nanoflowers. Besides the difference in mechanism, our nanocomposites also shows superior nanogenerator power density and stable piezocatalytic activity for re-usability (>90% dye-degradation efficiency is maintained even after using the same film 10 times).

## 2 Materials and methods

### 2.1 Synthesis of MoS_2_ nanoflowers

The MoS_2_ nanoflowers were synthesized by hydrothermal process. Typically, 0.28 g of Pluronic F-127 (Merck, Germany) was dissolved in 140 mL of distilled water. Next 8.5 g of Ammonium heptamolybdatetetrahydrate, (NH_4_)_6_Mo_7_O_24_.4H_2_O, (Merck, Germany) and 1.28 mg Thiourea, CH_4_N_2_S, (Merck, Germany) were added to the solution and allowed to stir at room temperature for 30 min. The resulting homogeneous solution was then loaded in a stainless-steel Teflon lined hydrothermal reactor and was kept in an oven at 200 °C for 24 h [12]. After the reaction, the black precipitate was centrifuged and washed with water and alcohol repeatedly. The precipitate was then dried to get a free-flowing powder and stored for further characterization and use.

### 2.2 Synthesis of MoS_2_-PVDF films

MoS_2_ nanoflower doped PVDF film was fabricated using solution processing. Initially, 250 mg of PVDF (Sigma-Aldrich, Germany; Mw: 180,000 GPC; Mn: 71000) was dissolved in 60 mL of Dimethyl sulfoxide (DMSO) (Merck, Germany) under constant magnetic stirring at 60 °C to obtain a homogeneous solution. Next, MoS_2_ (10 wt% of PVDF) powder was added to the PVDF solution and continuously stirred at room temperature until complete dispersion was achieved. Afterwards, the colloidal suspension was transferred to a clean Petri dish and dried at 80 °C in an oven to get nanocomposite films [22]. For comparison, pure PVDF film, without MoS_2_, was also prepared in a similar manner.

### 2.3 MoS_2_-PVDF nanogenerator (MPNG) fabrication

As a first application of the nanocomposite, a nanogenerator was fabricated by using an MoS_2_-PVDF film of dimension 1 cm × 1 cm × 50 μm. A 40 μm thick copper/aluminium electrode was attached on either sides of the film and two copper wires were connected to side of the film. Next, the film containing the electrodes and connecting wires are packaged in polydimethylsiloxane (PDMS) (Sylgard 184, Dow Corning, ratio of 1:10) by immersing the nanocomposite films in PDMS gel and drying for 15 min in vacuum followed by drying at 60 °C for 1 h to remove the bubbles from the mixture [22]. The final package size of the as-fabricated MPNG device was 2 cm × 2 cm × 0.3 cm.

### 2.4 Piezcatalysis experiment

MoS_2_-PVDF nanocomposite film of dimension 2 cm × 2 cm and thickness 50 μm was immersed in 10 mL of 10 ppm solutions of each dye (i.e., Acridine Orange (AO), Eosin Y (EO), Ethidium Bromide (ET) and Rhodamine B (RHO) (Loba chemie) in deionised water. The conical flask containing the dye solution and the nanocomposite film was placed in a bath sonicator (RS Pro Ultrasonic Cleaner, 100W) and subjected to pulsed ultrasonic vibrations for 20 min. During the experiment, a small portion of the dye solution was pipetted out at regular intervals (0, 5, 10, 15 and 20 min) and measured using UV–Visible spectrophotometer to check its degradation. The percent degradation and the rate constant were calculated from the measured data. A recyclability test was also performed with Rhodamine B which included 10 cycles of catalytic experiments using 200 mL of 10 ppm dye concentration to determine the efficacy of the nanocomposite films for treating large volumes of water. All the catalytic experiments were carried out in dark at 25 °C.

### 2.5 Instrumental

Physico-chemical characteristics including phase formation, microstructure, elemental distribution and thermal properties of MoS_2_ nanoflower and MoS_2_-PVDF film was performed using X-ray diffraction (XRD; Model-D8, Bruker AXS Inc., Madison, WI), Fourier transform infrared (FTIR) spectroscopy (FTIR-8400S, Shimadzu), Field emission scanning electron microscopy (FESEM; INSPECT F50, Netherlands), Energy dispersive x-ray analysis (EDAX) and Differential scanning calorimetry (DSC-60, Shimadzu). Ferroelectric properties of the film were measured in terms of polarization-electric field (P-E loop) using Hysteresis version 4.9.0 (Radiant technologies).

Open circuit voltage (V_oc_) generated by the MPNG under continuous finger imparting and ultrasound was measured using a digital storage oscilloscope (Keysight, Oscilloscope DSO-X 3012A). Short circuit current (I_sc_) was recorded under the same condition using Keysight, Electrometer B2985.

Catalytic degradation of dyes was monitored using a UV–Visible spectrophotometer (Lambda 650, PerkinElmer) and •OH was measured with terephthalic acid (Merck, Germany) using a Cary Eclipse Fluorescence spectrophotometer (Agilent Technologies).

## 3 Results and discussion

**Fig. 1:**
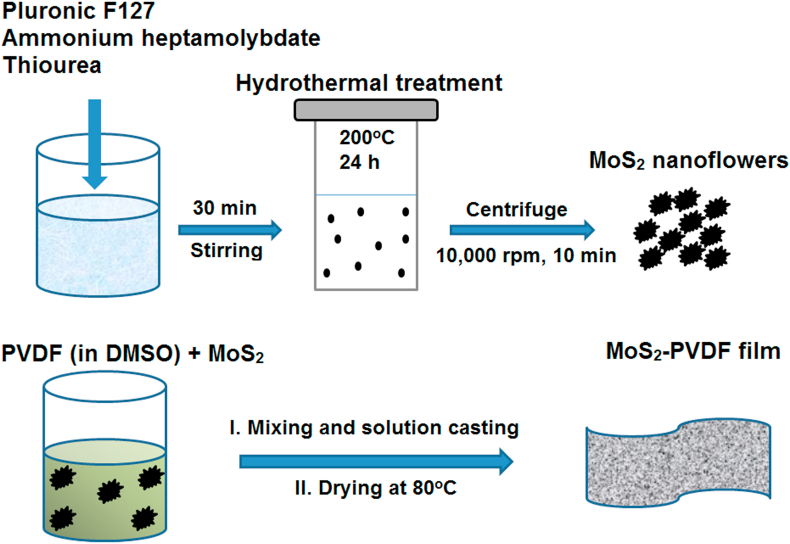
Scheme of MoS_2_-PVDF nanocomposite film preparation.

Fig. 1 shows the schematic of MoS_2_ and MoS_2_-PVDF nanocomposite synthesis procedure. The procedure described is a simple, water-based technique using Pluronic F-127 as the structure directing agent.

The role of F127 in promoting mesoporous flower like structure in γ-alumina is well documented by Ge et al. [32], and it is interesting to see that it also directs nanoflower morphology in case of MoS_2_. This provides an alternative, less expensive way than commonly used solvent like 1-Butyl-3-methylimidazolium chloride.

### 3.1 XRD analysis

**Fig. 2:**
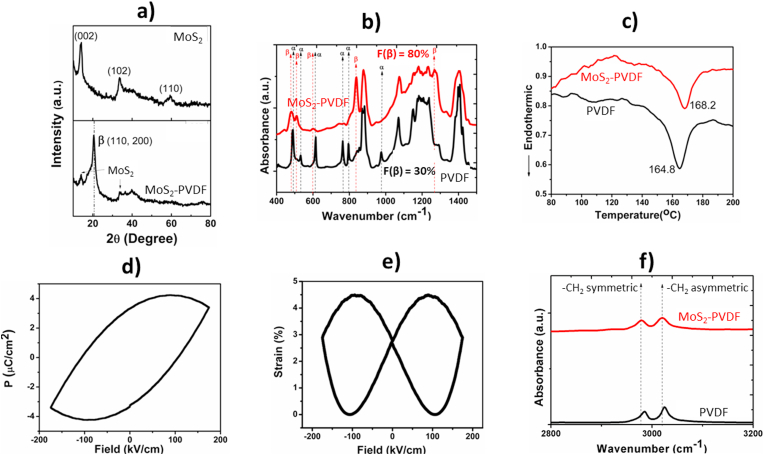
a) X-ray diffraction pattern of MoS_2_ and MoS_2_-PVDF film, b) FTIR spectra of the PVDF and the MoS_2_-PVDF films, c) DSC thermographs of the PVDF and the MoS_2_-PVDF films, d) P-E hysteresis loop, e) strain-field butterfly loop for the MoS_2_-PVDF film and f) FTIR spectra showing interaction of MoS_2_ with –CH_2_ backbone of PVDF.

The XRD pattern of pure MoS_2_ and MoS_2_-PVDF film is represented in Fig. 2a. MoS_2_ shows characteristic broad reflections at 13.97° (002), 33.66° (102), 39.97° (103), 49.94° (105) and 59.41° (110) which confirms phase formation in the as synthesized powder [11] (JCPDS card No. 37–1492). Addition of MoS_2_ nanoflowers significantly promotes nucleation of polar electroactive β-crystals in PVDF film which is indicated by a sharp single peak at 2θ = 20.5° ((110), (200)) and absence of α phase reflections. In addition, reflections corresponding to MoS_2_ are also present indicating the composite nature of the film (Fig. 2a). Electroactive β phase crystallization in PVDF is often achieved by introducing variety of molecules including ceramics, salts, clays, polymers, nanoparticles etc., whereby interfacial interaction with the C–H and fluorine groups leads to the all *trans* TTTT conformation [22,30,33]. In the present case, addition of MoS_2_ seems to be playing a similar role.

### 3.2 FTIR analysis

Comparison of FTIR spectra (Fig. 2b) of the pure MoS_2_ and the MoS_2_-PVDF films also indicates enhanced electroactive β phase formation in the composite film. Pure PVDF shows characteristic absorbance bands of nonpolar α-crystals at 488 cm^−1^ (CF_2_ waging), 532 cm^−1^ (CF_2_ bending), 615 and 764 cm^−1^ (CF_2_ bending and skeletal bending), and 796 and 976 cm^−1^ (CH_2_ rocking). A small peak at 840 cm^−1^ (CH_2_ rocking, CF_2_ stretching, and skeletal C–C stretching) is also observed in the spectrum of pure PVDF, which may be due to the slight presence of β or β + γ crystals (Fig. 2b). In case of MoS_2_-PVDF, all of the characteristic peaks corresponding to nonpolar α-crystals are absent with absorbance bands at 445 cm^−1^ (CF_2_ rocking and CH_2_ rocking), 479 cm^−1^ (CF_2_ deformation), 510 cm^−1^ (CF_2_ stretching), 600 cm^−1^ (CF_2_ wag), and 840 and 1274 cm^−1^ becoming predominant. The appearance of 510 cm^−1^ and 840 cm^−1^ band along with 445 cm^−1^ and 1274 cm^−1^ bands and the absence of the characteristic absorbance band of γ crystals at 1234 cm^−1^ confirms nucleation of electroactive β-crystals in the MoS_2_ doped PVDF film [28,30]. Presence of MoS_2_ was confirmed from the vibrations around 480 cm^−1^ (Mo–S stretching), 900 cm^−1^ (S–S stretching) and 1100 cm^−1^ (O–H stretching) which almost merges with the PVDF bands. The fraction of electroactive β phase content in the nanocomposite MoS_2_-PVDF film was calculated by using the Lambert-Beer Law

$$F\left(\beta \right)=\frac{A_{\beta }}{\left(\frac{K_{\beta }}{K_{\alpha }}\right)A_{\alpha }+\;A_{\beta }}$$

where A_α_ is the absorbance at 764 cm^−1^, A_β_ is the absorbance at 840 cm^−1^ and K_β_ equals 7.7 × 10^4^ cm^2^ mol^−1^ and K_α_ equals 6.1 × 10^4^ cm^2^ mol^−1^ as the absorption coefficients at 840 and 764 cm^−1^ respectively [22,33].

Using this equation, the β phase crystallization in pure PVDF is found to be 30% whereas for MoS_2_-PVDF it reached 80%. It is noted here that polar solvents like DMF, DMSO, DMAc can also induce β phase to certain extent in native PVDF by interacting with the chain but still require poling to exhibit piezoelectric properties [30]. Since the piezoelectric nature of PVDF is strongly dependent on the amount of electroactive β phase, therefore the addition of MoS_2_ nanoparticles clearly enhances its piezoelectric properties.

### 3.3 DSC thermograph analysis

The phase crystallization and melting behaviour of pure PVDF and MoS_2_-PVDF film was also analyzed by differential scanning calorimetry (DSC) to complement XRD and FTIR measurements. The melting peak at 164.5 °C in the DSC thermograph of pure PVDF film indicates presence of nonpolar α polymorph [22], whereas for the MoS_2_ doped PVDF, the melting peak is shifted to a higher temperature suggesting the nucleation of electroactive β phase in the nanocomposite film (Fig. 2c). The degree of crystallinity (X_c_) of the films have been evaluated by the formula X_c_ = ΔH_m_/ΔH_100%_, (where ΔH_m_ is the enthalpy of fusion of the samples and ΔH_100%_ is the enthalpy of fusion of 100% crystallite PVDF(104.6 J/g)) [22,33]. The enthalpy of fusion and crystallinity both increased upon MoS_2_ doping; MoS_2_-PVDF film had X_c_ ≈ 53.43% compared to X_c_ ≈ 33% for pure PVDF. This change is due to interaction between MoS_2_ nanoflowers and the polymer resulting in the formation of β-crystals in the nanocomposite film.

### 3.4 Ferroelectric characterization

Ferroelectric properties of MoS_2_-PVDF film were determined from the room-temperature polarization-electric field (P-E) hysteresis loop measurements at 50 Hz in the ±200 kV/cm range. The area within the loop corresponds to heterogeneous charge density and indicates charge storage capability of the material [22]. As can be seen from Fig. 2d, the MoS_2_-PVDF film shows a strong remnant polarization (Pr) value of 3.38 μC cm^−2^ compared to pure PVDF (0.038 μC cm^−2^ at 100 Hz). This high Pr value can be ascribed to good reversible ferroelectric behaviour and accelerated heterogeneous polarization tendency in the film. It also indicates that the material is inherently polar and piezoelectric in nature [22] since no poling was used in our synthesis process, confirming the self-poling property of these nanocomposite films. Following Katsouras et al. [34], the piezoelectric coefficient (d_33_) is calculated using the equation *d*_*33*_ *= -Pr/Y*, where Pr and Y is the remnant polarization and Young's modulus respectively at zero applied electric field (see ESI†). The Young's modulus of the MoS_2_-PVDF film was measured to be 928 N/mm^2^. Therefore, by using the above equation, d_33_ of MoS_2_-PVDFcomes out to be −36.4 pC/N. This value is higher than observed in poled PVDF [30] (−24 pC/N) showing that the composite film has superior piezoelectric properties without undergoing any poling process. The electrostriction coefficient Q_33_ was calculated to be 6.8 m^4^C^-2^ using the relation *d*_*33*_ *= Q*_*33*_*/2εε˳Pr*, where *ε* is the dielectric constant of the MoS_2_-PVDF film and *ε*˳ is permittivity of free space (8.854 × 10^−12^ F m^−1^). Based on Q_33_, longitudinal strain (S_3_) under polarization was calculated by using the equation *S*_*3*_ *= Q*_*33*_ *P*^*2*^. Using these strain values, a strain vs field curve was plotted (Fig. 2e) which shows a characteristic butterfly loop and confirms the reversible polarization and converse piezoelectric behaviour of the nanocomposite film [35].

**Fig. 3:**
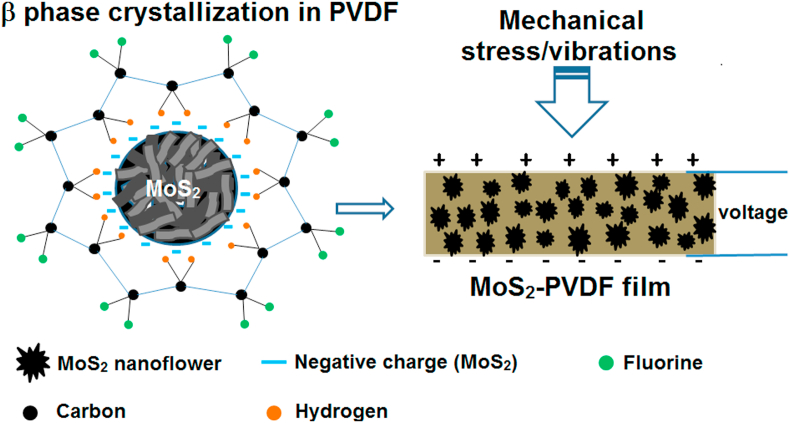
Mechanism of self-poling, β phase nucleation and the consequent mechanical stress induced piezoelectricity in the in the MoS_2_-PVDF nanocomposite film.

The above chemical, thermal and electromechanical characterisation establishes the self-poling feature of the MoS_2_-PVDF nanocomposite. In terms of microstructure, we can rationalise the observed self-poling property through either a possible hydrogen bonding or electrostatic interaction between Mo–S dipole in non-centrosymmetric MoS_2_ nanoflowers and positive-CH_2_ dipole of the polymer which induces β phase crystallization by orienting the polymer in a TTTT conformation as shown in Fig. 3.

Additionally, the –CF_2_ group may also interact with Mo in the S–Mo–S moiety if PVDF occupies interstitial position in MoS_2_ [28]. This interaction is further supported by a shift in the –CH_2_ asymmetric and symmetric stretching band vibrations in the high frequency (ῡ = 3050 − 2950 cm^−1^) region of FTIR (see Fig. 2f), which indicates interfacial interaction of MoS_2_ layers with –CH_2_/CF_2_ dipoles [28]. Furthermore, since MoS_2_ nanoflowers with abundant single and few layers exhibit strong piezoelectric potential at the edge sites, their incorporation in PVDF may also contribute to the overall enhanced piezoelectricity of the composite film through a synergistic effect [8,11]. This morphology dependent interaction of MoS_2_ is highlighted by the fact that primarily γ and α phase has been reported in MoS_2_ nanotube doped PVDF [36] while MoS_2_ nanosheets induced β phase in PVDF by synergistic electrostatic interaction [27,28]. Thus, in the present case, both inherent piezoelectricity and morphology of MoS_2_ nanoflower plays important role in the piezoelectric property of the composite. We note here that although our work clearly establishes the self-poled nature and the excellent piezoelectric features of the MoS_2_ nanoflower doped PVDF, which seem plausible based on molecular interactions and its characterisation, a more focussed investigation on mechanical and electrical characteristics of this nanocomposite and the role of nanoflower concentration will be required for a deeper understanding of the underlying physics.

### 3.5 FESEM imaging

**Fig. 4:**
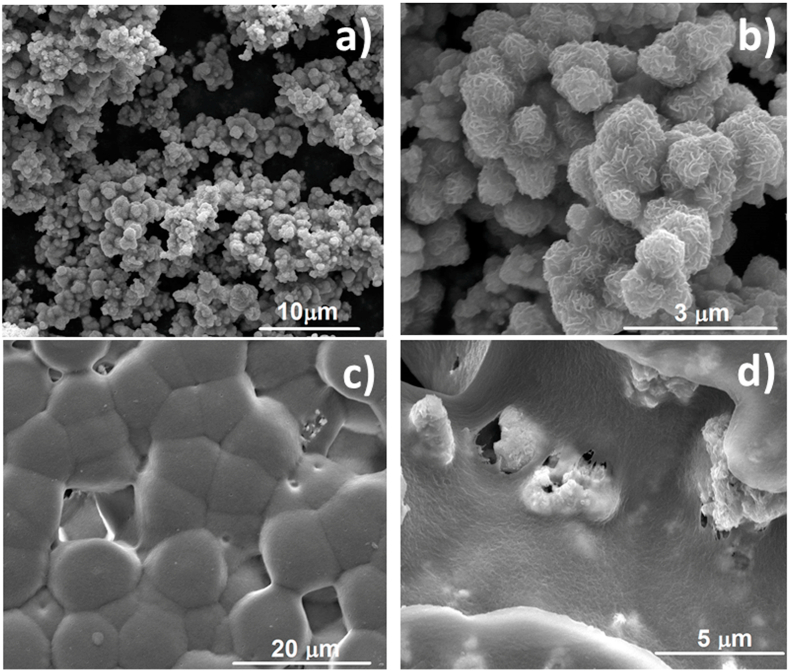
FESEM images of a) hydrothermally synthesized MoS_2_nanoflowers, b) magnified image of the nanoflowers showing abundant single and few layers MoS_2_, c) surface of MoS_2_-PVDF film showing spherullite formation due to β phase crystallization and d) fracture surface of a MoS_2_-PVDF film with embedded MoS_2_ nanoflowers.

The morphology of the piezoelectric films was assessed by FESEM (see Fig. 4). MoS_2_ particles are spherical in shape with particle size of ~0.8 μm (Fig. 4a). A magnified image (Fig. 4b) clearly shows that each particle has flower like morphology characteristic of single and few-layers MoS_2_ as described by Wu et al. [11]. Each nanoflower particle is in turn composed of typical aggregation of nano-petals having thickness of <100 nm (see Fig. S1a, ESI†).

MoS_2_ impregnated PVDF shows distinct formation of spherulites of diameter ~5–8 μm which confirms β phase crystallization [22] (Fig. 4c). The fracture surface of the MoS_2_-PVDF films shows the presence and distribution of MoS_2_ nanoflowers in the PVDF matrix (Fig. 4d). Most of the MoS_2_ is found to be embedded inside the polymer with very small fraction exposed to the environment at porous regions on the surface of the film. Although some local MoS_2_ particle aggregation exist, mostly the distribution is uniform in the PVDF matrix where the individual nanoflower particle morphology is maintained (Fig. S1b, ESI†).

Elemental mapping (Fig. S2, ESI†) and EDAX (Fig. S3, ESI†) was also performed to analyse the distribution of MoS_2_ particles in the PVDF matrix. A homogeneous distribution of particles was observed throughout the film which indicates uniform dispersion and interaction with PVDF.

### 3.6 MoS_2_-PVDF nanogenerator (MPNG) performance

**Fig. 5:**
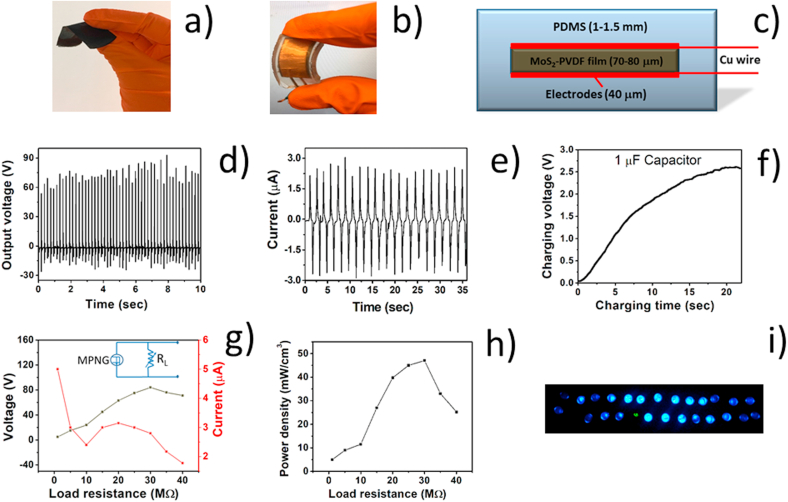
a) Flexible MoS_2_-PVDF film, b) nanogenerator prototype (i.e. the MPNG), c) schematic showing MPNG fabrication design. Performance of MPNG under continuous human finger tapping, d) open circuit voltage (V_oc_), e) short circuit current (I_sc_), f) capacitor charging, g) variation of voltage and current across with different load resistances (1 MΩ–40 MΩ), inset shows the corresponding circuit diagram, h) effective power density variation with different load resistances (1 MΩ–40 MΩ) and i) powering up commercial LED bulbs.

Fig. 5 captures the nanogenerator functionality of the MoS_2_-PVDF film (Fig. 5a). The device flexibility and layered construction of the package are shown in Fig. 5b and c, respectively. Both the film and the prototype nanogenerator device (see Fig. 5b) are highly flexible and robust, showing excellent energy harvesting capability from mechanical energy. The piezoelectric output of the MPNG device under periodic human finger tapping (~5 Hz) is shown in Fig. 5d. An impressive open circuit voltage (V_oc_) of 84V is obtained under a tapping force of 27.5 N and the corresponding short circuit current (I_sc_) reached a value of 3.05 μA (Fig. 5e).

The variation of the output voltage from MPNG was further evaluated across different load resistances (ranging from 1 to 40 MΩ); the instantaneous voltage increased and gradually reached a peak value of 84 V at 30 MΩ similar to open voltage circuit (V_oc_) (Fig. 5g). The corresponding instantaneous current (I_L)_ is calculated by the equation I_L_ = V/R_L_, where, V is the voltage measured across load resistance R_L_. The effective power density is determined from equation P = (V^2^/R_L_) 1/v, where, v is the effective volume (surface area × thickness). The device produced an outstanding peak power density of 47.14 mW cm^−3^ at 30 MΩ load (Fig. 5h). To the best of our knowledge, this is the highest power density obtained by finger tapping in MoS_2_ nanoparticle doped PVDF which again highlights the synergistic piezoelectric effect in our self-poled nanocomposite film. The observed high power density can be rationalised for two reasons. First, the contributions due to the strain induced piezoelectric potential generated by the self-poled PVDF and the MoS_2_ nanoflowers, under vigorous tapping force (~27 N), reinforce each other. Second, we surmise that the vigorous tapping also causes additional strain induced spontaneous polarization and consequent dipole formation leading to charge separation (electron and hole pairs) and accumulation at the abundant edge sites of piezoelectric MoS_2_ nanoflowers and PVDF interface [8,11,28]. This should enhance the piezoelectric output. A comparison of the device with other previously reported PVDF based nanogenerators is summarized in ESI† (Table S1). The pure PVDF based nanogenerator only produced 4 V under the same tapping force (see ESI†, Fig. S4). The as fabricated nanocomposite film also produced an output of 2V in an ultrasonic bath (100 W) (see ESI†, Fig. S5) which shows that it is highly sensitive over a wide range of frequency.

The overall possible working mechanism of the device is depicted in Fig. 3. Under mechanical stress, the polarization produced (by separation of charges) in the piezoelectric film (see Fig. 3) can be harnessed in terms of electric power by using electrodes on either side of the film which drives the electrons through the external circuit. When the stress is released, the electrons flow in the opposite direction and a negative potential is reached [[8], [9], [10], [11],^28^,^35^]. To demonstrate the application potential of the as fabricated MPNG, it was connected to a 1 μF capacitor through a bridge rectifier. As shown in Fig. 5f, the capacitor is charged very swiftly to 2.5 V within 20 s under finger tapping (see Table S2, ESI† for comparison). Further, the device was able to power up 25 commercial LEDs when connected in series through a full wave bridge rectifier (Fig. 5i and video S1, ESI†) showing that they can be promising candidates for developing portable self-powered electronic and medical devices.

### 3.7 Piezocatalytic dye degradation

**Fig. 6:**
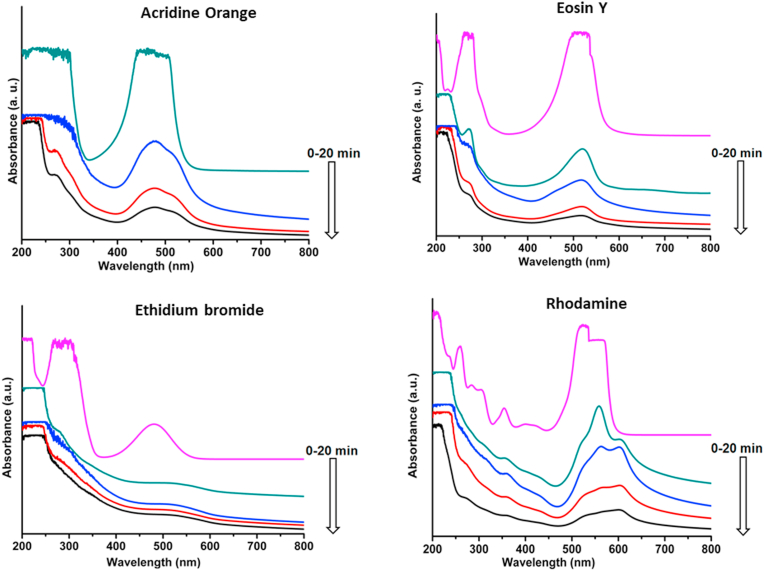
UV–Visible spectra of dyes undergoing piezocatalytic degradation under ultrasonic vibration in dark- Acridine orange (top left), Eosin Y (top right), Ethidium bromide (bottom left) and Rhodamine B (bottom right).

Piezocatalytic dye degradation was monitored using UV–Visible spectroscopy. As observed from Fig. 6, the dyes showed rapid degradation within 20 min of sonication with the nanocomposite MoS_2_-PVDF film in dark condition. The degradation can be visually observed (see Fig. S6, ESI†) from the change in dye colour after 25 min of ultrasonication thereby clearly demonstrating their piezocatalytic property. In contrast, pure PVDF film failed to engender any appreciable degradation of dyes even after prolonged sonication (50 min) (Fig. S7, ESI†).

**Fig. 7:**
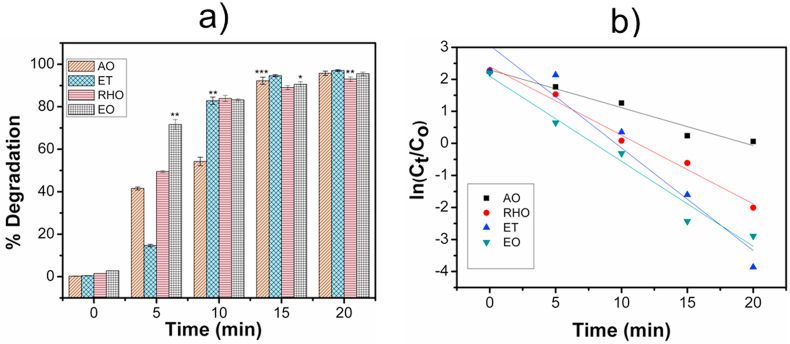
a) Variation in dye degradation by MoS_2_-PVDFfilm and b) the rate of degradation over time. C_o_-initial concentration (10 ppm), C_t_-concentration at time t. Values are ± standard error of the mean of five experiments done in triplicate (one way ANOVA). *p > 0.05, *p > 0.005 and *p > 0.0005.

The percent degradation over time is presented in Fig. 7a, where the MoS_2_-PVDF film shows excellent catalytic activity in dark with >90% degradation achieved for each dye. However, with undoped PVDF film, the amount of dyes remained unchanged even after prolonged ultrasonication which indicates that the piezoelectric effect in MoS_2_-PVDF film is playing a major role in the catalytic process. The degradation reaction followed pseudo first order kinetics as indicated from the rate of degradation curve (Fig. 7b). The highest rate constant was achieved for Ethidium bromide (ET) (0.32 min^−1^) followed by Eosin Y (0.26 min^−1^), Rhodamine B (0.21 min^−1^) and Acridine Orange (0.127 min^−1^), respectively. To date, degradation of these dyes has been mainly limited to using photocatalytic nanoparticles (ZnO, TiO_2_, SnO_2_, Fenton's reagent, Au and Ag etc) under light irradiation [[22], [23], [24], [25],[37], [38], [39], [40], [41]]. In contrast, in the present case this is achieved by MoS_2_-PVDF film through a fast-piezo driven catalytic process which harnesses vibrational energy to degrade dyes under dark conditions, which are more suited to industrial effluents flowing through pipelines. Although MoS_2_ nanoflowers – in powder form – has been reported to degrade Rhodamine B [8,10,11] but this is the first time a nanocomposite is shown to piezocatalytically degrade potentially carcinogenic dyes like Ethidium bromide, Eosin Y and Acridine Orange.

**Fig. 8:**
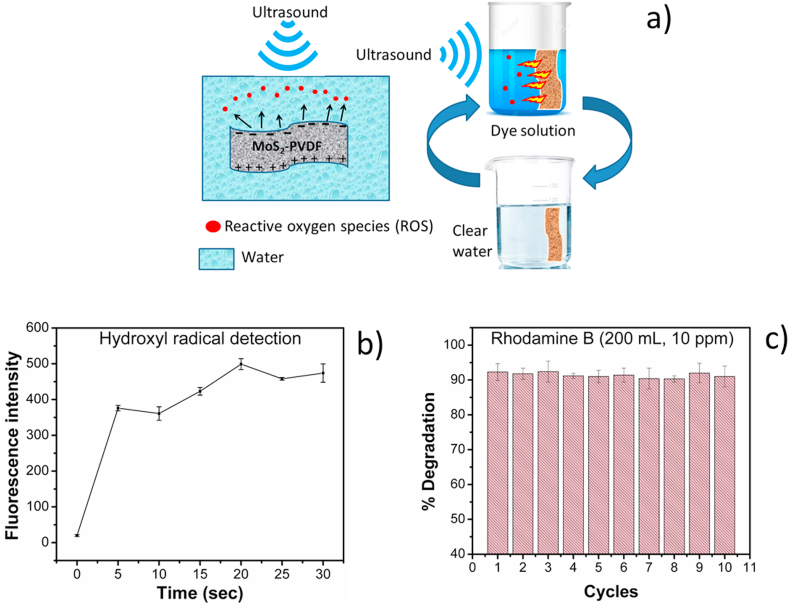
a) Mechanism of piezocatalysis of the MoS_2_-PVDFfilm, b) hydroxyl radical evolution under ultrasonic bath vibration and c) re-usability test of MoS_2_-PVDF film showing stable degradation of Rhodamine B for 10 cycles.

Based on this observation, a piezocatalytic mechanism of dye degradation is proposed as presented in Fig. 8 below. As already mentioned, incorporation of MoS_2_ nanoflowers converts an otherwise unpoled piezoelectric PVDF to a self-poled and highly piezoelectric nanocomposite. Since MoS_2_ is already piezocatalytic, therefore its inclusion in the PVDF matrix results in boosting the piezoelectric as well as piezocatalytic property of the overall nanocomposite. Thus, when the piezoactive MoS_2_-PVDF film is subjected to ultrasonic vibrations in an aqueous solution containing dye, MoS_2_ nanoflowers are mechanically agitated to generate electron-hole pairs by spontaneous polarization of MoS_2_ nanoflowers. This polarization is further enhanced by the interaction of MoS_2_ with PVDF where piezoelectric β phase is induced. These electron and hole pairs then migrate (in opposite direction) and accumulate to the film surface aided by the dispersed MoS_2_ particles in PVDF. Continuous rapid vibration by ultrasound generates and accumulates enough electrons and holes at the film surface to split water form ROS like ^•^OH, ^•^O^−^ etc. Hence, the organic dye molecules are rapidly degraded by the produced ROS similar to a photocatalytic process [8,10,11] (Fig. 8a). However, it may be mentioned here that surface of the film (SEM image in Fig. 4d) contains scattered porous regions where MoS_2_ is partially exposed to the dye solution and thus will contribute to dye degradation as well.

Lin et al. [31], reported piezocatalytic activity, where they used MoS_2_ nanoflower-PDMS to degrade Rhodamine B in running water. However, the piezocatalytic activity solely came from exposed MoS_2_ on the PDMS surface and showed deterioration in activity with time with time due to spillage when the composite is subjected to friction with water. In case of our MoS_2_-PVDF nanocomposite however, as explained by XRD, FTIR and SEM, since MoS_2_ actively induces β phase by interacting with the PVDF chain. We believe that this interaction also leads to a more robust attachment between MoS_2_ and PVDF compared to PDMS, at the loading concentration used (i.e, 10 wt.% MoS_2_). A time lapse photo of the film immersed in the Rhodamine B dye solution and subjected to ultrasonic vibration is presented in Fig. S8 (ESI†) showing gradual decolorization of the water without any apparent degradation of the film over a period of 25 min. However, when the film was loaded with 20% (w/w) MoS_2_, the water turned greyish due to spillage of the excess MoS_2_ particles, while showing much faster degradation of dyes as already reported by Wu et al. [8,11]*.*

The mechanical stimulus driven generation of ^•^OH radical in the dye solution is confirmed by trapping it with terephthalic acid at regular intervals under ultrasonics agitation. Terephthalic acid forms a fluorescent hydroxyterephthalic acid upon reaction with ^•^OH radical, which offers qualitative means to detect the radical formation with high sensitivity [42]. From Fig. 8b, it can be observed that ^•^OH radical is being gradually generated with time after ultrasound exposure and reaching a stable value around 20 min which coincides with the catalytic degradation time. One of the main advantages of using MoS_2_-PVDF films in water purification would be their reusability since the nanocomposite is inherently piezocatalytic and mechanically robust. In order to assess this feature, the piezocatalysis experiment was repeated using the same film with fresh solution of Rhodamine B (200 mL, 10 ppm). The result presented in Fig. 8c shows stable catalytic performance over 10 cycles with >90% efficiency using 200 mL of dye solution under dark conditions. However, since the volume is large the degradation time was over 40 min. This also confirms the robustness of our PVDF based nanocomposite film in contrast to the work of Lin et al. [31], where the catalytic activity decreased with progressive cycle (67% at 4th cycle) due to release of MoS_2_ particles from PDMS surface. FESEM of the film surface post experiment (Fig. S9, ESI†) shows that structural integrity is maintained even after repeated use. Thus, the developed piezocatalytic film provides safe and reliable production of on-demand ROS which may be applied to degrade ecotoxic organic and inorganic pollutants from water reservoirs, drainage and sewer systems where photocatalytic application is not feasible or is difficult to implement.

## 4 Conclusion

This work reports the synthesis of a highly robust, efficient and self-poled piezoelectric nanocomposite and applies it to two key areas namely water remediation and energy harvesting. The nanocomposite showed strong piezocatalytic activity as demonstrated by a rapid and efficient degradation (>90%) of some potentially carcinogenic dyes under ultrasound vibrations within a short time of 20 min. Such catalytic reaction is found to be initiated by production of reactive oxygen species in aqueous solution as a result of strong piezoelectric effect by the nanocomposite film under dark condition as opposed to photocatalysis which requires light. The origin of piezoelectricity in the nanocomposite film was studied in detail by using different analytical and electrical measurements to conclude that MoS_2_ nanoflowers play a vital role in inducing the β crystals in PVDF which result in spontaneous orientation of dipoles leading to self-poled piezoelectricity in the nanocomposite film. Also, the overall functionality of the nanocomposite is enhanced due to entrapment of inherently piezoelectric and piezocatalytic MoS_2_ nanoflowers in the polymer matrix. The films are robust and reusable and showed promise of purifying large volume of dye contaminated water (200 mL) over 10 cycles using only vibrational energy. In addition, the nanocomposite has been successfully exploited as an efficient energy harvesting device (nanogenerator) which can be activated by mechanical stimuli from common human and natural activities. The nanogenerator produced a maximum output voltage of 84 V and a power density of 47.14 mW cm^−3^ under human finger tapping and powered up 25 LED lights. Overall, the development of such advanced and versatile piezoelectric nanocomposite materials opens up a novel, energy-efficient way to novel water remediation technologies and smart self-powered electronic devices.

## CRediT authorship contribution statement

**Biswajoy Bagchi:** Conceptualization, Visualization, Methodology, Formal analysis, Investigation, Writing - original draft, Writing - review & editing. **Nur Amin Hoque:** Methodology, Validation, Formal analysis. **Norbert Janowicz:** Methodology. **Sukhen Das:** Resources. **Manish K. Tiwari:** Conceptualization, Supervision, Visualization, Project administration, Formal analysis, Validation, Resources, Funding acquisition, Writing - review & editing.

## Declaration of competing interest

The authors declare the following financial interests/personal relationships which may be considered as potential competing interests: The corresponding author this manuscript is a director of an UCL spin out company (TCR Materials Ltd) which is trying to commercialize superhydrophobic materials. We do not perceive this to be a conflict.
