# Supplementary material for: Re-usable self-poled piezoelectric/piezocatalytic films with exceptional energy harvesting and water remediation capability
Source: Nano Energy. 2020 Dec;78:105339. doi: 10.1016/j.nanoen.2020.105339 (PMC8417815; doi:10.1016/j.nanoen.2020.105339)
Supplement: Multimedia component 1 [file mmc1.docx]

**Electronic Supplementary Information**

**Re-usable, self-poled piezoelectric/piezocatalytic films with exceptional energy harvesting and water remediation capability**

Biswajoy Bagchi^1,2^, Nur Amin Hoque^3^, Norbert Janowicz^2^, Sukhen Das^3^ Manish K. Tiwari*^1,2^

**^1^**Wellcome/EPSRC Centre for Interventional and Surgical Sciences, UCL, London, W1W 7TS, UK

**^2^**Nanoengineered Systems Laboratory, Mechanical Engineering, UCL, London, WC1E 7JE, UK

**^3^**Jadavpur University, Department of Physics, Kolkata,700032, India

*Corresponding author

E-mail: [m.tiwari@ucl.ac.uk](mailto:m.tiwari@ucl.ac.uk), Contact: +442031081056

**SEM of MOS_2_ nanoflower and MOS_2_-PVDF nanocomposite film**


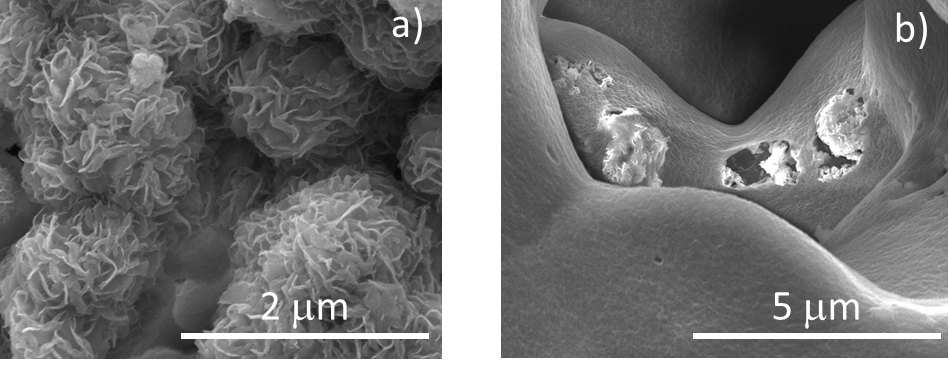


Figure S1: SEM micrographs showing a) microstructure of MoS_2_ nanoflower particles which are composed single or few layers of nanopetals and b) distribution of nanoflower particle as observed on the surface of the MOS_2_-PVDF film.

**Elemental mapping and EDAX spectra of MoS_2_-PVDF**


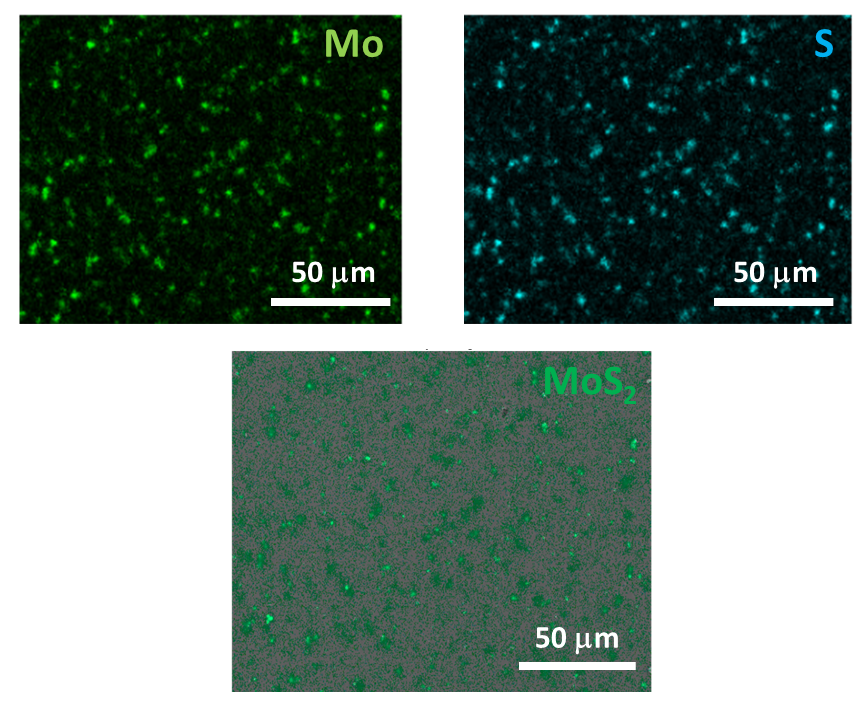


Figure S2: Elemental mapping of the surface of MoS_2_-PVDF piezoelectric film showing distribution of molybdenum (Mo, top left image), Sulphur (S, top right image) and overlapped image (below) of MoS_2_ as a whole.


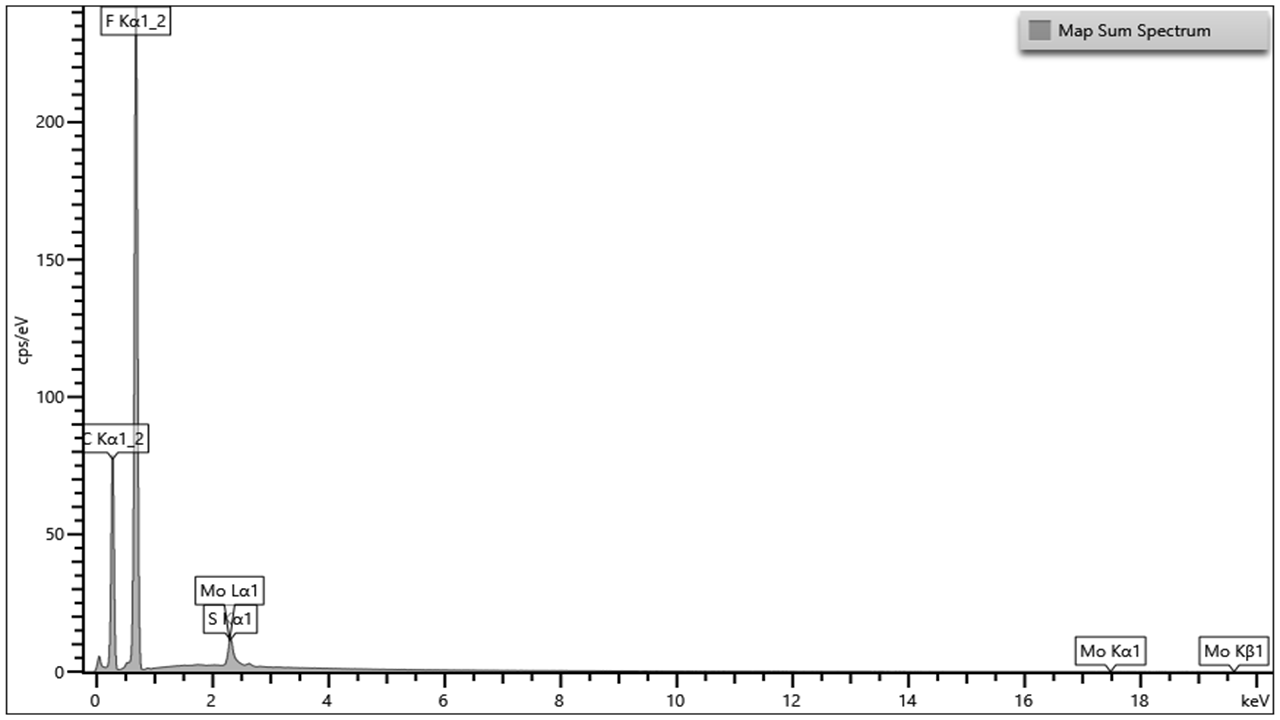


Figure S3: EDAX spectrum of MoS_2_-PVDF film surface showing relative concentration of MoS_2_ in the PVDF matrix.

**Calculation of the piezoelectric coefficient d_33_**

The d_33_ value was calculated following the commonly used ‘dimensional model’ as reported by Katsouras et al. (ref. [34] in the main manuscript):

$d\text{33}=\frac{\partial P\text{s}}{\partial\sigma}\text{|}\begin{matrix} \text{ } \\ \text{ } \\ \text{E=0} \end{matrix} = \frac{\partial\left( \frac{\mu}{V} \right)}{\partial\sigma}= P\text{r} \left( \frac{\partial ln\mu}{\partial\sigma}-\frac{\partial lnV}{\partial\sigma} \right)= P\text{r} \left( \frac{\partial ln\mu}{\partial\sigma}-\frac{1}{Y} \right) \approx- \frac{P\text{r}}{Y}$ (1)

where P_s_ is the spontaneous polarization due to macroscopic dipole moment µ, V is the sample volume, σ applied stress, P_r_ the remnant polarization and Y the Young’s modulus of the film.

Here the $\frac{\partial ln\mu}{\partial\sigma}$ is insignificant and is neglected assuming rigidity of the dipoles, and the compliance $\frac{\partial lnV}{\partial\sigma}$ , *by definition*, is (1/Y), i.e. the inverse of Young’s modulus (see ref. [34] in the main manuscript).

Thus, according to this model and assumptions, d_33_ can be expressed as a ratio of P_r_ and Y. We determined P_r_ from P-E loop measurements and measured Y separately.

**Calculation for external force (F) on the MPNG**

The applied force on the MPNG during finger tapping can be qualitatively estimated following momentum and energy conservation law: $mgh=\frac{mv^{2}}{2}$, i.e.,

$v=\left( 2gh \right)^{\frac{1}{2}}$ (2)

$\left( F-mg \right)\Delta t=mv$ (3)

where *m*, *v*, *h* and *F* represent the mass of the impacting object, i.e. finger(s), velocity of the object when it touches the MPENG, falling height of the object and applied force, respectively. The symbol *g* denotes acceleration due to gravity and *Δt* is the time between two successive positive or negative peak of the voltage versus time graph.

In present work, *m* = 1.7 kg, *v* = 1.384 m/s, *h* = 0.15 m (determined through video recording, see Video S1), and *g* = 9.8 m/s^2^, *Δt* = 0.2167 s. Thus, using equation (3), the force of the finger impacts on the MPNG is determined to be *F* = 27.5 N which corresponds to a pressure of 64.5 kPa.


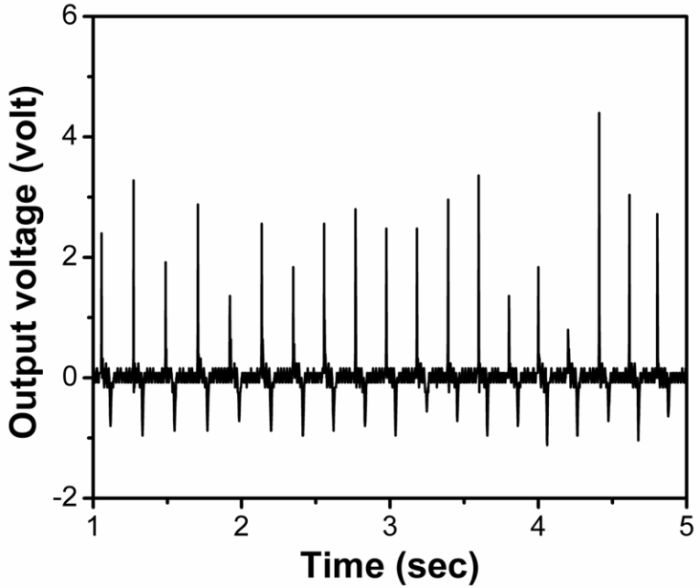


Figure S4: Open circuit output voltage of pure PVDF based nanogenerator under human finger tapping force which shows a weak output voltage due to some amount of piezoelectric phase developed during solution casting process.

**
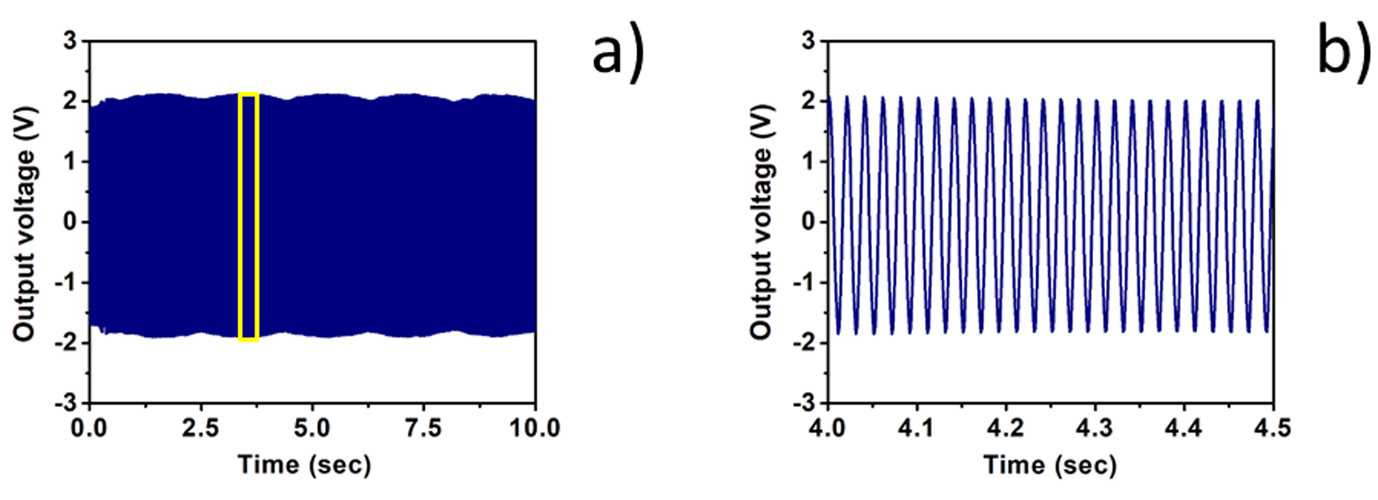
**

Figure S5: Open circuit output voltage of MPNG under a) ultrasonic vibration and b) magnified image from the selected region of a) (marked by yellow rectangle) showing individual peaks.

Table S1**:** Comparison of output voltage, current and power density of our MPNG with some previously reported nanogenerators based on PVDF nanocomposites. Our self-poled, MPNG device shows highest achievable performance under human finger tapping based on voltage output, current, power density and force applied.

| **Nanogenerator** | **Poling**  **Status** | **Voltage**  **(V)** | **Current**  **(I)** | **Power**  **Density** | **Pressure** | **Dimensions**  **(Area ×thickness)** |
| --- | --- | --- | --- | --- | --- | --- |
| PVDF/AlO-rGO^[1]^ | Self-poled | 36 V | 0.8 μA | 27.97 μW/ cm^3^ | 36 kPa | 7.82 cm^2^ ×  0.13 cm |
| rGO-Ag/PVDF^[2]^ | Self-poled | 18V | 1.08 µA | 28 µW/cm^3^ | NA | 22 cm^2^ × N.A. |
| Fe-rGO/PVDF^[3]^ | Self-poled | 5.1V | 0.254 µA | N.A. | 12 kPa | 6 cm^2^ × N.A. |
| ZnO NWs/PVDF^[4]^ | 100 kV/mm | 0.2 V | 10 nA | 2 μW/cm^3^ | N.A. | N.A. |
| Nano ZnO/PVDF (0.2M ZnO loading)^[5]^ | 5 MV/m | 4 V | N.A. | N.A. | 4.4 kPa | 0.25 cm^2^ × 10 µm |
| Cerium complex-PVDF^[6]^ | Self-poled | 36 V | N.A. | N.A. | N.A. | 3.75 cm^2^ × N.A. |
| γ-PVDF/ZnO^[7]^ | Self-poled | 28 V | 450 nA | 0.4 µW/cm^3^ | 8.43 kPa | 7.2 cm^2^ × N.A. |
| PVDF/DNA^[8]^ | Self-poled | 20 V | 0.184 μA | 11 μW/cm^2^ | 63 kPa | N.A. |
| BaTiO_3_-PVDF^[9]^ | 2 kV | 35 V | 600 nA | NA | 1 MPa | 1 cm^2^ × N.A. |
| FAPbBr_3_-PVDF^[10]^ | 50 kV/cm | 30 V | 6.2 μA | 27.4 μW/cm^2^ | 0.5 MPa | 1.68 cm^2^ × N.A. |
| PVDF/ 2D-MoS_2_^[11]^ | 10 kV | 14V | N.A. | N. A. | 8.8 kPa | N.A. |
| PVDF/few layer MoS_2_-cellulose^[12]^ | 18 kV | 50 V | 30 nA | N.A. | N.A. | 9 cm^2^ × N.A. |
| PVDF/ZnO (*in situ*)^[13]^ | Self-poled | 50 V | 3.05 µA | 32.8 mW/cm^3^ | 70 kPa | 0.64 cm^2^ × 20 µm |
| Niobate/PVDF^[14]^ | Self-poled | 18V | 2.6 µA | N.A. | 125 kPa | 4 cm^2^ × N.A. |
| **Present work** | **Self-poled** | **84V** | **2.8 µA** | **47.14 mW/cm^3^** | **67.5 kPa** | **1 cm^2^× 50 µm** |

Table S2**:** Comparison of capacitor charging ability of our MPNG with other PENGs reported earlier.

| **Nanogenerator** | **Capacitor (μF)** | **Time required (s)** |
| --- | --- | --- |
| PVDF/AlO-rGO^[1]^ | 2.2 | 96.6 |
| Cerium complex-PVDF^[6]^ | 1 | 70 |
| γ-PVDF/ZnO^[7]^ | 1 | 100 |
| PVDF/DNA film^[8]^ | 2.2 | 230 |
| FAPbBr_3_-PVDF^[10]^ | 3.3 | 200 |
| PVDF/ 2D-MoS_2_^[11]^ | 1 | 44 |
| PVDF/ZnO (*in situ*)^[13]^ | 1 | 13 |
| Yb^3+^/ PVDF^[15]^ | 1 | 30 |
| P(VDF–HFP)/Zn^2+^ film^[16]^ | 1 | 70 |
| **Present work** | **1** | **20** |

**Piezocatalytic effect of MoS_2_-PVDF**

**
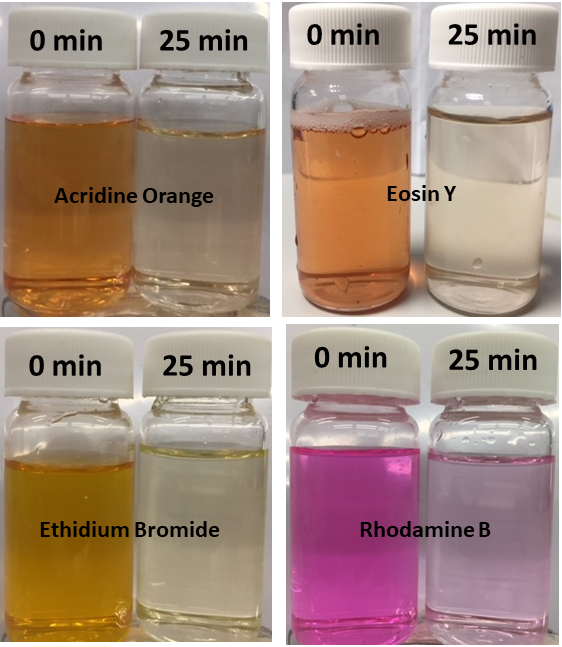
**

Figure S6: Piezocatalytic degradation of dyes as observed by relative decolourization after 25 minutes of ultrasonic vibration with PVDF-MoS_2_ film.


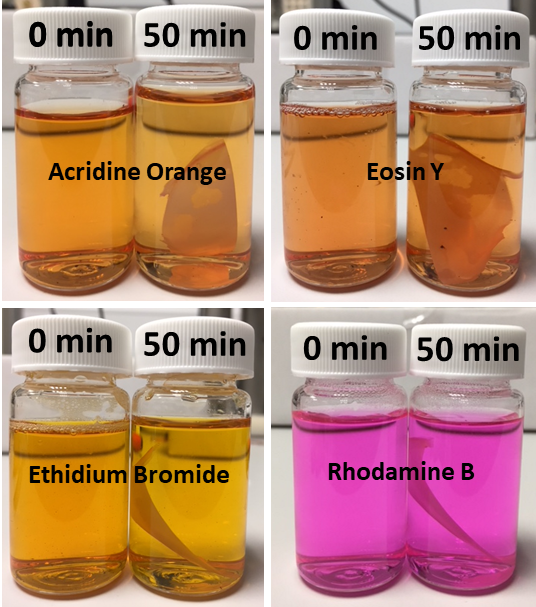


Figure S7: Assessing degradation of dyes by pure PVDF film (without any MoS_2_) under ultrasonic vibration. Clearly there is no dye degradation.


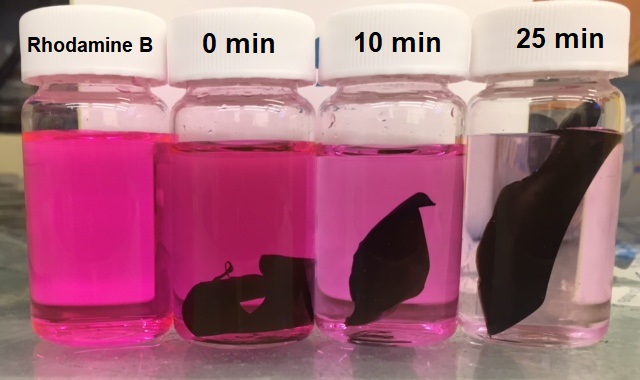


Figure S8: Time dependent piezocatalytic degradation of Rhodamine B by MoS_2_-PVDF films under ultrasonic vibration.


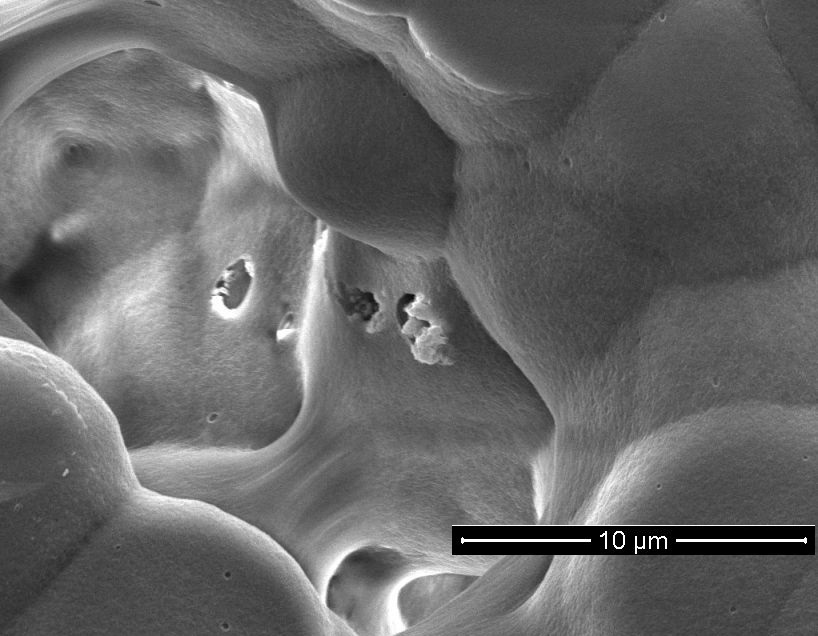


Figure S9**:** SEM micrograph of MoS_2_-PVDF film surface after 10 cycles of catalytic experiment showing negligible degradation of microstructure.

Video S1**:** Demonstration of lighting up commercially available blue LEDs using our MPNG under finger tapping.

Video S2: Demonstration of output voltage from the MPNG device under gentle finger tapping.

Video S3: Demonstration of maximum output voltage from the MPNG device under vigorous finger tapping.

Video S4: Demonstration of output voltage from the MPNG device under bending and twisting motion (showing negligible triboelectric effect).

**References:**

[1] S. K. Karan, R. Bera, S. Paria, A. K. Das, S. Maiti, A. Maitra, B. B. Khatua, An approach to design highly durable piezoelectric nanogenerator based on self‐poled PVDF/AlO‐rGO flexible nanocomposite with high power density and energy conversion efficiency, Adv. Energy Mater. 6 (2016) 1601016-1601028.

[2] L. Pusti, L. Sinha, P. M. Shirage, A flexible self-poled piezoelectric nanogenerator based on a rGO–Ag/PVDF nanocomposite, New J. Chem. 43(2019) 284-294.

[3] S. K. Karan, D. Mandal, B. B. Khatua, Self-powered flexible Fe-doped RGO/PVDF nanocomposite: an excellent material for a piezoelectric energy harvester, Nanoscale, 7(2015)10655-10666.

[4] M. Lee, C. Y. Chen, S. Wang, S. N. Cha, Y. J. Park, J. M. Kim, L. J. Chou, Z. L. Wang, A hybrid piezoelectric structure for wearable nanogenerators, Adv. Mater. 24(2012) 1759-1764.

[5] R. Bhunia, S. Das, S. Dalui, S. Hussain, R. Paul, R. Bhar, A. K. Pal, Flexible nano-ZnO/polyvinylidene difluoride piezoelectric composite films as energy harvester, Appl. Phys. 122(2016) 637-650.

[6] S. Garain, T. K. Sinha, P. Adhikary, K. Henkel. S. Sen, S. Ram, C. Sinha, D. Schmeiber, D. Mandal, Self-poled transparent and flexible UV light-emitting cerium complex−PVDF Composite: A high-performance nanogenerator, ACS Appl. Mater. Interfaces 7(2015)1298−1307.

[7] S. Jana, S. Garain, S. K. Ghosh, S. Sen, D. Mandal, The preparation of γ-crystalline non-electrically poled photoluminescant ZnO–PVDF nanocomposite film for wearable nanogenerators, Nanotechnology, 27(2016)445403-445415.

[8] A. Tamang, S. K. Ghosh, S. Garain, M. M. Alam, J. r. Haeberle, K. Henkel, D. Schmeisser, D. Mandal, DNA-assisted β-phase nucleation and alignment of molecular dipoles in PVDF Film: A realization of self-poled bioinspired flexible polymer nanogenerator for portable electronic devices, ACS Appl. Mater. Interfaces, 7(2015) 16143-16147.

[9] Y. Zhao, Q. Liao, G. Zhang, Z. Zhang, Q. Liang, X. Liao, Y. Zhang, High output piezoelectric nanocomposite generators composed of oriented BaTiO3 NPs@PVDF Nano Energy, 11(2015) 719-727.

[10] R. Ding, X. Zhange, G. Chenc, H. Wangd, R. Kishorb, J. Xiaod, F. Gaob, K. Zengd, X. Chenc, X. W. Sune, Y. Zheng, High-performance piezoelectric nanogenerators composed of formamidinium lead halide perovskite nanoparticles and poly(vinylidene fluoride), Nano Energy, 37(2017) 126-135.

[11] K. Maity, B. Mahanty, T. K. Sinha, S. Garain, A. Biswas, S. K. Ghosh, S. Manna, S. K. Ray, D. Mandal, Two‐dimensional piezoelectric MoS_2_‐modulated nanogenerator and nanosensor made of poly(vinlydine fluoride) nanofiber webs for self‐powered electronics and robotics, Energy Technol. 5(2017) 234-243.

[12] P. Sahatiya, S. Kannan, S. Badhulika, Few layer MoS_2_ and in situ poled PVDF nanofibers on low cost paper substrate as high performance piezo-triboelectric hybrid nanogenerator: Energy harvesting from handwriting and human touch, Appl Mater Today, 13(2018)91-99.

[13] P. Thakur, A. Kool, N. A. Hoque, B. Bagchi, F. Khatun, P. Biswas, D. Brahma, S. Roy, S. Banerjee, S. Das, Superior performances of in situ synthesized ZnO/PVDF thin film based self-poled piezoelectric nanogenerator and self-charged photo-power bank with high durability, Nano Energy, 44(2018) 456-467.

[14] C. Zhang, Y. Fan, H. Li, Y. Li, L. Zhang, S. Cao, S. Kuang, Y. Zhao, A. Chen, G. Zhu, Z. L. Wang, Fully Rollable Lead-Free Poly(vinylidene fluoride)-Niobate-Based Nanogenerator with Ultra-Flexible Nano-Network Electrodes, ACS Nano 12 (2018) 4803−4811.

[15] S. K. Ghosh, A. Biswas, S. Sen, C. Das, K. Henkel, D. Schmeisser, D. Mandal, Yb^3+^ assisted self-polarized PVDF based ferroelectretic nanogenerator: A facile strategy of highly efficient mechanical energy harvester fabrication Nano Energy, 30(2016)621-629.

[16] P. Adhikary, D. Mandal, Enhanced electro-active phase in a luminescent P(VDF–HFP)/Zn^2+^ flexible composite film for piezoelectric based energy harvesting applications and self-powered UV light detection, Phys.Chem.Chem.Phys**.** 19(2017)17789-17798.
